# The Potential of iPS Cells in Synucleinopathy Research

**DOI:** 10.1155/2012/629230

**Published:** 2012-03-18

**Authors:** Leonhard Linta, Marianne Stockmann, Tobias M. Boeckers, Alexander Kleger, Stefan Liebau

**Affiliations:** ^1^Institute for Anatomy and Cell Biology, Ulm University, 89081 Ulm, Germany; ^2^Department of Internal Medicine I, Ulm University, 89081 Ulm, Germany

## Abstract

*α*-synuclein is a protein involved in the pathogenesis of several so-called synucleinopathies including Parkinson's disease. A variety of models have been so far assessed. Human induced pluripotent stem cells provide a patient- and disease-specific model for *in vitro * studies, pharmacotoxicological screens, and hope for future cell-based therapies. Initial experimental procedures include the harvest of patients' material for the reprogramming process, the investigation of the patients genetic background in the cultured cells, and the evaluation of disease-relevant factors/proteins under various cell culture conditions.

## 1. ***α***-Synuclein and Parkinson's Disease

*α*-synuclein is a protein that is believed to interact with presynaptic vesicles and to be involved in the regulation of dopamine transport, secretion, and reuptake [1], possibly by interacting with the SNARE complex [2]. In addition, it is believed to have additional nuclear and cytoplasmic functions. However it gained its prominence through its role in the pathology of Parkinson's disease (PD) and several other neurodegenerative diseases summarized as synucleinopathies [3]. *α*-synuclein can form fibrils and *α*-synuclein containing aggregates, so-called Lewy bodies and Lewy dendrites which are major characteristics of PD neuropathology. Their involvement in PD occurrence and neurodegeneration has not yet been finally unraveled. The discovery of *α*-synuclein overexpressing or point mutations in some PD patients, which have a higher tendency to form fibrils, additionally strengthened the belief that this protein has one of the central roles in PD [4]. Several *in vitro *and *in vivo *models have therefore been established to study the formation of fibrils, Lewy Bodies, and the mechanism of neurodegeneration [5]. Interestingly, the high tendency to form fibrils seems to be characteristic for the human protein. While mice overexpressing mouse *α*-synuclein had no neural phenotype, mice overexpressing the human *α*-synuclein suffered from neurodegeneration [6]. This showed that although protein function and interactions as well as pathologic mechanisms could be partly analyzed in animals and cultured animal cells, all these findings have to be carefully checked in a human system which is as close to the real disease pathology as possible. In addition, the use of human cells could even reveal additional mechanistical findings that could not be mimicked in rodents.

## 2. Disease Models in Synucleopathies

Basically, disease modeling is performed in several branches. In brief, *In vivo *studies include primarily patient's clinical features associated with disease morphology and progress [7, 8]. Clinical studies for PD include the evaluation of, for example, radiological changes (organ morphology, transmitter release/uptake, signs of degeneration such as plaques or metabolic dysfunction), symptom rating, disease course, or pharmacotoxicological trials. Secondly, genetic investigations searching for disease relevant gene abberations and familiar cosegregation are of great value for the understanding and treatment of such degenerative syndromes. Additionally, *in vivo *modeling includes several animal models starting from lower animals such as the worm *C. elegans*, the fly *D. melanogaster* or the Zebrafish *D. rerio* [9, 10]. These kinds of animal models not only provide systematic insights into genetic disease background but also help to elucidate pathways in pathogenesis. Apart from that, they allow a relatively easy to handle model at low costs. Still, diseases like PD also involve studies using higher animals up to models mimicking the human organism, namely, monkeys and apes. Certainly, most higher animal models consist of mice models in various compositions [5]. These mice can nowadays be generated relatively easy with valuable features such as genetic knock down or even organ specific and/or in an inducible manner. These models are utilized for a variety of studies. Mouse models in general stand for investigations of, for example, the pathomechanisms, disease progression, gene function, or pharmacotoxicological evaluations. On the other hand, *in vitro *studies often make use of cellular setups. Of high interest for PD studies are cell cultures consisting of dopaminergic neurons from different sources. Until recently, most of these cell models were harvested from rodents or other animals. Investigations on these models carry the advantage of broad access to detailed cellular mechanisms. Genetic modulation of single cells additionally provide insights into cellular processes such as differentiation, migration, and function or degenerating processes such as apoptosis or necrosis. Especially, stem cells are used for studies of differentiation and maturation. In PD several different sources and types of stem cells are used. The following exemplary differences exist: (i) pluripotent embryonic stem (ES) cells are a good source for dopaminergic neurons and may be used for future cell therapeutic approaches and as platforms for pharmacotoxicological assays. Still, they inherit ethical and legal prohibitions and harbor certain dangers such as teratoma formation *in vivo*. (ii) Neural stem cells (adult stem cells) provide a source for even autologous dopaminergic neurons and can be used for patient-specific and disease-specific pathogenic investigations [11–15]. Nevertheless, these cells are extremely difficult to harvest, and this is only possible by harmful surgical intervention. Additionally, (up to now) these cells cannot be passaged over a long time and lose their potential to generate dopaminergic neurons over time. (iii) Mesenchymal stem cells from the bone marrow are thought to be amongst the most easy to harvest individual stem cell sources. These cells are also thought to be a certain source for dopaminergic neurons and provide a good hope for future cell-based therapies for a variety of neurodegenerative disorders [16]. But, the efficiency of dopaminergic differentiation is very low and research is still far away from a cell-based therapy.

## 3. Induced Pluripotent Stem Cells as a Disease Model

**Figure 1:**
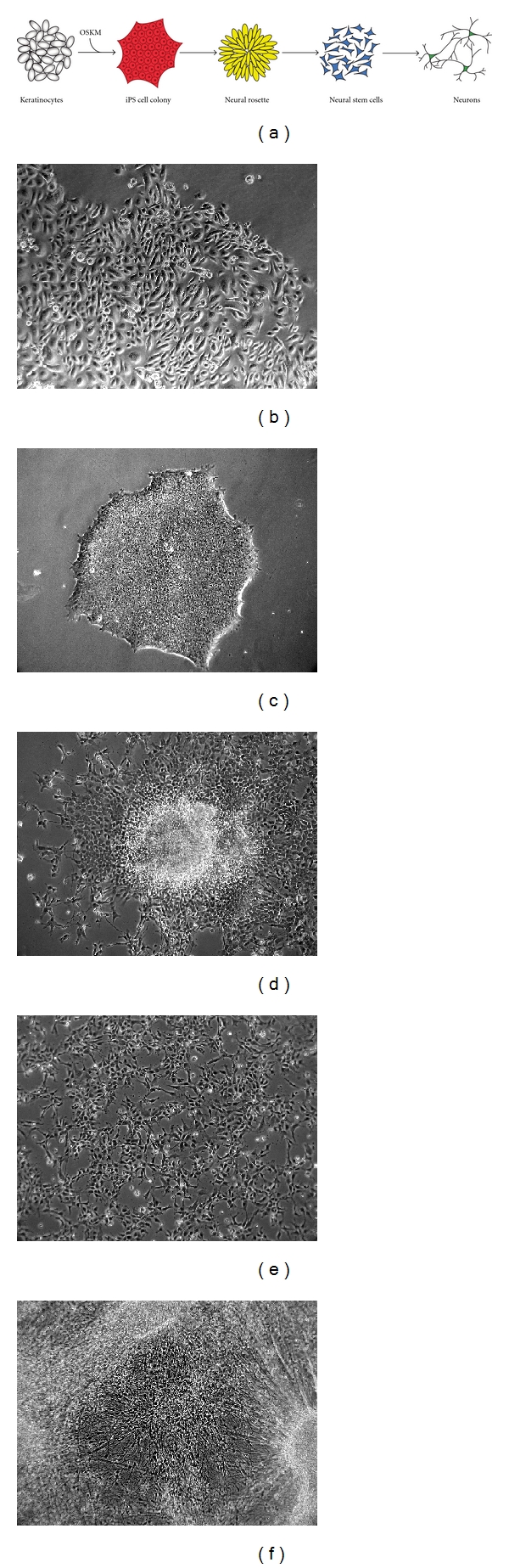
Production of iPS cell-derived neurons follows basic developmental steps. (a) Keratinocytes are reprogrammed into iPS cells by forced overexpression of the Yamanaka factors (OSKM). After differentiation into ectodermal cells neural stem cells can be isolated from the centers of the forming neural rosettes. These neural stem cells can be subsequently differentiated into neurons, (b) proliferating keratinocyte culture, (c) iPS cell colony in a feeder-free culture, (d) neural rosette shortly before dissection and isolation of neural stem cells, (e) adherent culture of neural stem cells, and (f) iPS cell-derived neurons after four weeks of culture.

Studying neurodegenerative diseases in human cells is of course a difficult task. Since the affected cells cannot be propagated in culture and the supply of primary material is very limited, they cannot be widely used as a model system. As depicted in the last paragraph the use of ES cells and the subsequent differentiation into neural stem cells and neurons could partially circumvent this barrier. However their use is discussed very controversially in several countries due to ethical concerns. This issue has been resolved by the discovery of induced pluripotent stem cells (iPS cells). iPS cells are produced from somatic cells like fibroblasts or keratinocytes and can be reprogrammed by the forced overexpression of certain transcription factors (known as the Yamanaka factors Oct4, Sox2, Klf4, c-Myc (OSKM)) into a state that strongly resembles embryonic stem cells [17]. These cells can subsequently be subjected to differentiation into virtually all cells of the organism [18] and of course to neural differentiation (as depicted in Section 4 in more detail) (Figure 1(a)), especially into dopaminergic neurons which are most affected by PD [19]. This method could prove even more valuable by the use of cells from PD patients with *α*-synuclein mutations to evaluate and compare their iPS cell-derived neurons with healthy ones. By these means it is possible to verify findings from animal cell culture systems and other *in vitro* assays in human cells very similar or even identical to the ones which are actually affected in PD patients. Therefore it is of importance to establish a variety of iPS cell lines from different donors with *α*-synuclein-related diseases.

## 4. Generation and Differentiation of Patient-Specific iPS Cells

The first question when reprogramming somatic cells into iPS cells is that of the cell type being reprogrammed. Traditionally, most groups used fibroblasts from punch biopsies since they are relatively easy to get and to propagate. However, when planning to generate patient-specific cell lines one has to consider that the acceptance to perform a punch biopsy is not very high since it is still an invasive and painful process. Therefore we favor the use of keratinocytes from plucked scalp hair as a starting cell source (Figure 1(b)). These cells can be obtained by noninvasive means and, in addition, have a much higher reprogramming efficiency compared to skin fibroblasts [18, 20]. Recent findings indicating that redifferentiating iPS cells favor cell types close to their origin before reprogramming reinforce the benefits of keratinocytes as starting cells since they are of ectodermal origin and closer related to neurons than fibroblasts [21]. 

The delivery of the four reprogramming factors Oct4, Sox2, Klf4 and c-Myc is preferably done via lentiviral transfection of a polycistronic and excisable construct. This system still harbors the highest efficiencies [22, 23]. Although there were several other methods described, including transient transfection or protein transduction [24, 25], these have very low efficiencies and are not well usable for the generation of patient-specific cell lines. The transfection of modified RNAs was described as very efficient for reprogramming but has still to be evaluated on a broader basis [26]. Lentiviral transfection of course has the negative effect of random DNA integration into the genome. This can partially be diminished by using cre-excisable lentiviral constructs. However, in order to minimize side effects caused by the integration as well as the variances between different lines it is important to evaluate a certain number of lines, preferably from different donors.

When producing patient-specific iPS cells it is important to have high reprogramming efficiencies, since the patient material is limited. Therefore the use of high-quality cultures of the reprogrammed cells as well as the feeder cells used in the reprogramming process is crucial. In addition several selection methods have been described to ease the isolation of true iPS cells [27, 28]. The arising iPS cells have to be thoroughly characterized to ensure their true iPS cell identity (Figure 1(c)).

The differentiation of iPS cells into neurons has already been extensively studied with ES cells [29, 30]. Available protocols, although greatly varying in detail, share some general steps. Typically, differentiation of iPS cells is started by withdrawal of FGF2. In suspension culture this is used to form embryoid bodies containing precursor cells of all lineages. The differentiation into the ectodermal and neuroectodermal lineage can, however, be highly enhanced by addition of the BMP antagonist Noggin (as well as the small molecule dorsomorphin) and even more in combination with SB431542, a TGF*β* pathway inhibitor [31, 32]. Together these two substances can induce strong neural differentiation even under adherent conditions and in lines with a low neural differentiation potential. Under adherent conditions cells start to form neural rosettes (Figure 1(d)). They consist of PAX6 or Nestin-positive neural stem cells (NSCs) and mimic the development of the neural tube *in vitro*. To exclude undifferentiated cells or cells differentiating into a different fate the inner regions of the neural rosettes can be mechanically or enzymatically detached. This ensures a high purity and a similar differentiation stage of the NSCs. NSCs can be cultured under adherent conditions or in suspension as neurospheres (Figure 1(e)). However, it is not clear for how long these cells can be cultured without a reduction or change in their differentiation potential. Different culture conditions for NSCs and thereafter of the arising neurons have been reported to favor the generation of certain neuronal subtypes, like glutamatergic neurons, dopaminergic neurons, or motor neurons (Figure 1(f)) [30, 33]. To induce final differentiation cells are treated with a mixture neurotrophic factors like the brain-derived or the glial-derived neurotrophic factor (BDNF and GDNF) as well as region-specific morphogens like Sonic hedgehog. High reproducibility in cell survival, culture quality and synapse formation has been reported for cocultures with glial cells [34]. These could be of mouse or human origin but also generated themselves from patient-specific iPS cells [35]. Of course this could be of relevance especially for diseases where glial cells cause or contribute to the pathologic effects.

## 5. Perspectives in iPS Cell-Based Synucleinopathy Research

**Figure 2:**
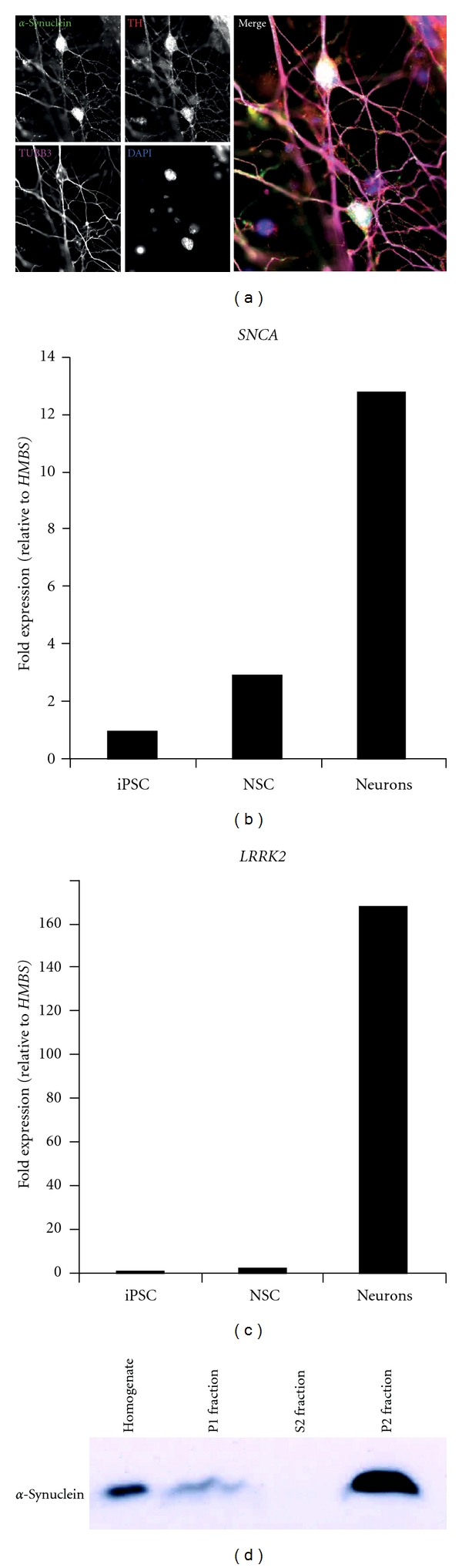
iPS cell-derived neurons express *α*-synuclein. (a) Immunofluorescence stainings of *α*-synuclein in TH (tyrosine hydroxylase) and TUBB3 (Tubulin beta-III) positive dopaminergic neurons after 5 months of differentiation show nuclear and vesicular localisation of *α*-synuclein, (b) and (c) RNA expression of *α*-synuclein (*SNCA*) and leucine-rich repeat kinase 2 (*LRRK2*) is upregulated in neurons compared to iPS cells and neural stem cells (NSCs) (normalized to the house-keeping gene *HMBS*), and (d) Subcellular fractionation of iPS cell-derived neurons shows nuclear (P1 fraction) and membrane associated, likely synaptic localization (P2 fraction) of *α*-synuclein.

The aim of upcoming iPS cell-based studies would be to study the morphology and electrophysiological behavior of synucleinopathy-derived neurons and compare them with healthy cells. Since *α*-synuclein is especially involved in the synaptic compartment, alterations there would be of great interest [2]. It was already shown that iPS cell-derived human neurons express *α*-synuclein [36]. The first synuclein-related patient iPS cell line-derived neurons with a triplication of the *α*-synuclein (*SNCA*) gene show a higher amount of *α*-synuclein protein compared to healthy control cells [37]. In addition to the already published relatively young neurons, we could show *α*-synuclein in immunostainings of mature iPS cell-derived neurons with a nuclear as well as vesicular staining pattern (Figure 2(a)). They also express the gene at a higher rate compared to iPS cells or NSCs (Figure 2(b)). Interestingly, the gene *LRRK2* (leucine-rich repeat kinase 2) is also upregulated in differentiated neurons (Figure 2(c)). This PD-associated gene was described to enhance the ability of *α*-synuclein to form aggregates [38]. Neurons derived from patient iPS cells with a LRRK2 mutation show enhanced stress sensitivity and an elevated *α*-synuclein levels [39]. In a subcellular fractionation *α*-synuclein is present in the nuclear fraction (P1) but mainly in the P2 fraction containing membrane associated proteins and the synaptic compartment (Figure 2(d)). Another very intriguing study would be to evaluate ageing in these iPS cell-derived neurons. For this they have to be kept in culture for prolonged periods of time and/or additionally stressed to provoke the formation of plaque-like structures *in vitro*. This would be a very powerful tool since it then would recapitulate the neuronal changes observed in PD patients. If cultured cells can be reliably provoked to form *α*-synuclein aggregates and plaques they also would be an ideal readout system for pharmaceutical research and evaluation of potential PD drugs. Since solely human *α*-synuclein and its mutated forms seem to have this high tendency to form plaques the use of iPS cell-derived human neurons can be crucial to evaluate the exact pathomechanisms involved in formation of synucleinopathies. The final step would be to recapitulate the observed phenotypes of the patient-derived cells in healthy cells where the genes of interest are artificially modified. This method has already been demonstrated with *α*-synuclein point mutations [40]. The additional use of such isogenic controls with single alterations is important to finally prove the monogenic disease potential of genes like *α*-synuclein or LRRK2 and rule out additional but yet unknown mutations.

##  Conflict of Interests

The authors declare no potential conflicts of interest.
